# Acute pancreatitis with colon cutoff sign

**DOI:** 10.1002/ccr3.8086

**Published:** 2023-10-19

**Authors:** Ryo Shinomiya, Naonori Inoue, Takuji Kawamura, Koji Uno

**Affiliations:** ^1^ Department of Gastroenterology Kyoto Second Red Cross Hospital Kyoto Japan

**Keywords:** acute pancreatitis, colon cutoff sign, CT scout images

## Abstract

**Key Clinical Message:**

Acute pancreatitis can present with a colon cutoff sign. The colon cutoff sign can also occur in gastric cancer, splenic artery bleeding, and ruptured abdominal aortic aneurysm. A CT scout image can also be an important laboratory finding for diagnosing a disease.

**Abstract:**

A 22‐year‐old woman visited our hospital with a complaint of epigastric pain. An abdominal contrast‐enhanced computed tomography (CT) scan revealed that intestinal gas was interrupted at the splenic flexure on the CT scout image (colon cutoff sign). Scan images showed a poorly contrasted area in the pancreatic tail. Based on these results, the patient was diagnosed with acute pancreatitis. The colon cutoff sign is an image showing the spread of inflammation to the colon. A CT scout image can also be an important laboratory finding for diagnosing a disease.

## Graphical abstract

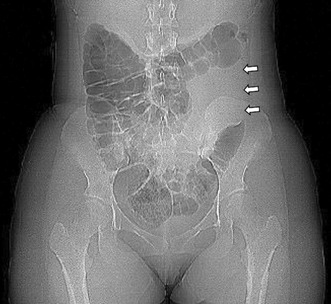

## 1 CLINICAL CASE

**FIGURE 1:**
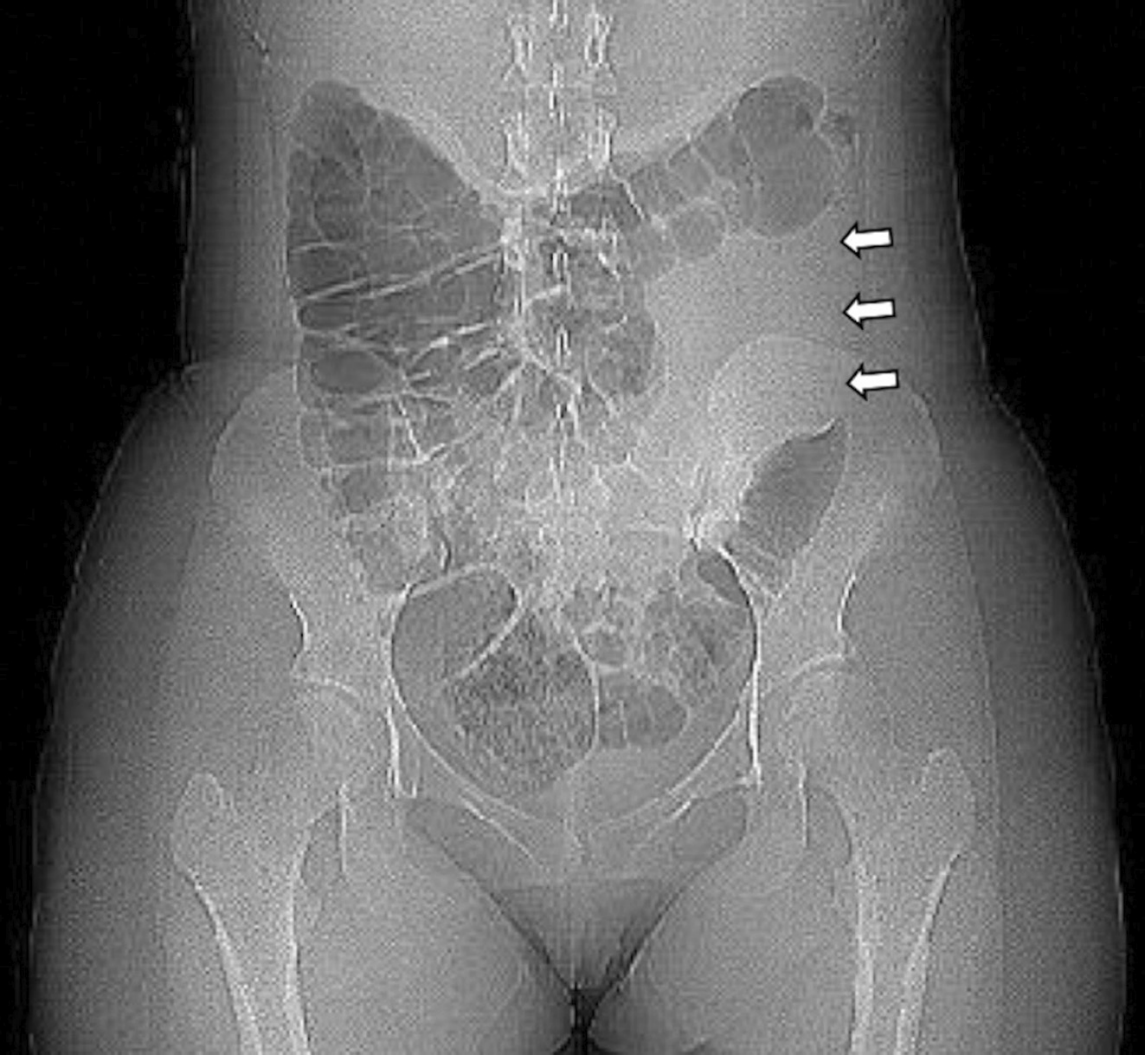
CT scout image. Intestinal gas is interrupted from the splenic flexure to the middle of the descending colon (arrow).

**FIGURE 2:**
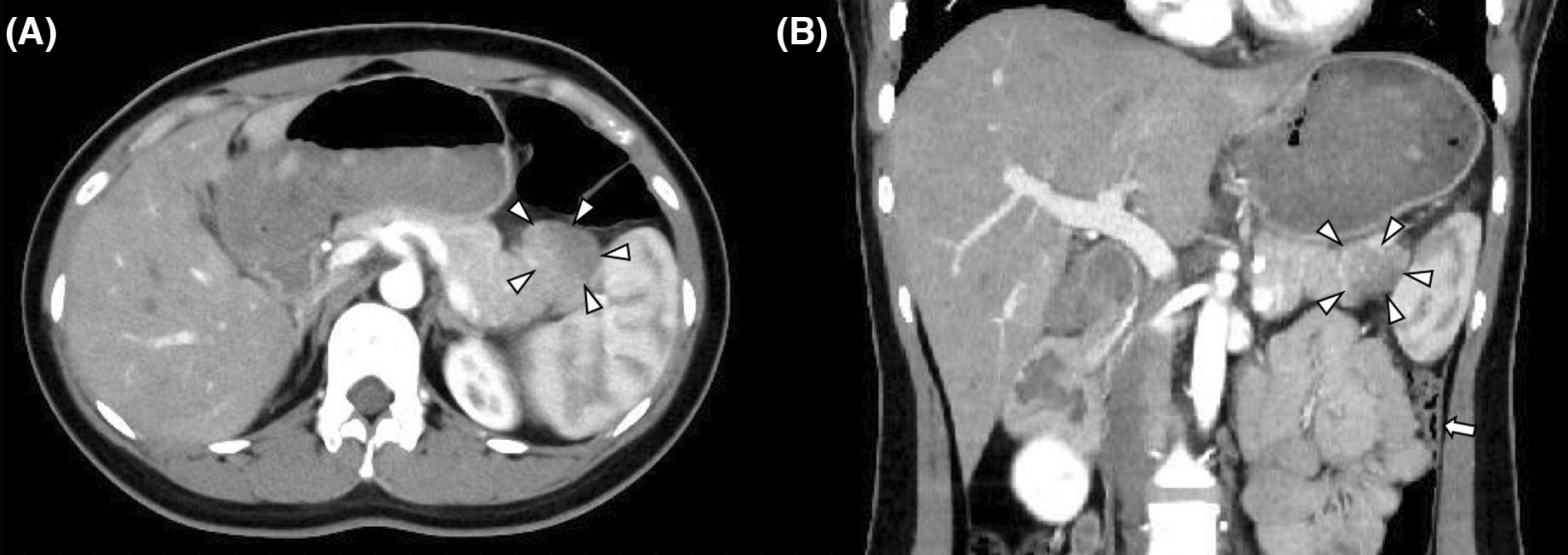
Abdominal intravenous contrast‐enhanced CT. (A) Axial section (B) Coronal section. Poor contrast area in the tail of the pancreas (arrowhead). Narrowing of the descending colon (arrow).

A woman in her 20s with no medical history visited our hospital with a complaint of epigastric pain. Her vital signs at the time of consultation were as follows: body temperature, 37.0°C; pulse, 92 beats/min; blood pressure, 113/71 mmHg; and SpO_2_, 96%. Her abdominal findings were flat, soft, epigastric pain, and diminished bowel peristaltic sounds. A blood test revealed the following: white blood cells (WBC) 7200/μL; C‐reactive protein (CRP), 0.21 mg/dL; amylase 802 U/L; and lipase, 2182 U/L. An abdominal contrast‐enhanced computed tomography (CT) scan revealed that intestinal gas was interrupted at the splenic flexure on the CT scout image (Figure 1). Scan images showed a poorly contrasted area in the pancreatic tail (Figure 2). Increased adipose tissue concentration and fluid retention were observed near the same area. Based on these results, the patient was diagnosed with acute pancreatitis. She did not have excessive alcohol intake or gallstones, and she was diagnosed with idiopathic acute pancreatitis. After she was hospitalized, treatment began with infusions and protease inhibitor administration. Pancreatic enzymes, which showed high levels, gradually improved, and epigastric pain also improved. A MRI scan was performed to confirm the presence of gallstones that could not be detected by CT, but no stones were found. She was in good shape and was discharged 9 days after admission.

A colon cutoff sign on abdominal plain X‐ray is a sign that is associated with acute pancreatitis. In 1956, Price reported three cases of acute pancreatitis in which localized gas retention was observed from the ascending colon to the hepatic curve, but the gas image was not visible on the anal measurement of the hepatic curve. This was called the colon cutoff sign.^1^ The most common site is the splenic flexure, and the importance of the phrenicocolic ligament has been suggested as the reason for this phenomenon. This ligament connects directly or indirectly from the splenic flexure to the gastrosplenic ligament, gastrocolic ligament, and splenocolic ligament. Acute pancreatitis causes inflammatory substances, including pancreatic enzymes, to leak around the colonic splenic flexure through the phrenicocolic ligament and the aforementioned ligaments. Thus, intestinal tract spasm and paralytic ileus occur. Continued effects of pancreatic enzymes cause colonic ischemia, leading to intestinal edema. The sensitivity of the colon cutoff sign ranges from 2% to 52% and varies widely. This may be due to differences in the definition of the colon cutoff sign and the severity of pancreatitis.^2^ In some cases, severe inflammation leads to perforation of the large intestine after pancreatitis. The frequency of intestinal perforation due to acute pancreatitis is 0.8%–8%. The colon cutoff sign is an image showing the spread of inflammation to the colon, and the possibility of subsequent colon perforation should also be considered. The colon cutoff sign can also occur in gastric cancer, splenic artery bleeding, and ruptured abdominal aortic aneurysm.^3^ CT scout images can also be an important laboratory finding when testing for acute abdomen.

## CONSENT

Written informed consent was obtained from the patient to publish this report in accordance with the journal's patient consent policy.

## AUTHOR CONTRIBUTIONS

**Ryo Shinomiya:** Investigation. **Naonori Inoue:** Conceptualization; writing – original draft. **Takuji Kawamura:** Conceptualization. **Koji Uno:** Conceptualization.

## Data Availability

The data that support the findings of this study are available from the corresponding author upon reasonable request.
